# Intuition and Moral Decision-Making – The Effect of Time Pressure and Cognitive Load on Moral Judgment and Altruistic Behavior

**DOI:** 10.1371/journal.pone.0164012

**Published:** 2016-10-26

**Authors:** Gustav Tinghög, David Andersson, Caroline Bonn, Magnus Johannesson, Michael Kirchler, Lina Koppel, Daniel Västfjäll

**Affiliations:** 1 JEDILab, Division of Economics, Department for Management and Engineering, Linköping University, Linköping, Sweden; 2 The National Center for Priority Setting in Health Care, Department of Medical and Health Sciences, Linköping University, Linköping, Sweden; 3 Department of Economics, Norwegian School of Economics, Bergen, Norway; 4 Department of Economics, Stockholm School of Economics, Stockholm, Sweden; 5 Department of Clinical and Experimental Medicine, Linköping University, Linköping, Sweden; 6 Department of Behavioural Sciences and Learning, Linköping University, Linköping, Sweden; 7 Department of Banking and Finance, University of Innsbruck, Innsbruck, Austria; 8 Centre for Finance, Department of Economics, University of Gothenburg, Gothenburg Sweden; 9 Decision Research, Eugene, Oregon, United States of America; University of the Basque Country, SPAIN

## Abstract

Do individuals intuitively favor certain moral actions over others? This study explores the role of intuitive thinking—induced by time pressure and cognitive load—in moral judgment and behavior. We conduct experiments in three different countries (Sweden, Austria, and the United States) involving over 1,400 subjects. All subjects responded to four trolley type dilemmas and four dictator games involving different charitable causes. Decisions were made under time pressure/time delay or while experiencing cognitive load or control. Overall we find converging evidence that intuitive states do not influence moral decisions. Neither time-pressure nor cognitive load had any effect on moral judgments or altruistic behavior. Thus we find no supporting evidence for the claim that intuitive moral judgments and dictator game giving differ from more reflectively taken decisions. Across all samples and decision tasks men were more likely to make utilitarian moral judgments and act selfishly compared to women, providing further evidence that there are robust gender differences in moral decision-making. However, there were no significant interactions between gender and the treatment manipulations of intuitive versus reflective decision-making.

## Introduction

A key issue in moral psychology is to what extent moral decisions are governed by fast, automatic, and intuitive “system 1” processes or by slower, more controlled, and reflective “system 2” processes [1–4]. Utilitarianism and its behavioral offspring *Homo Economicus* are founded on the idea that decisions are based on deliberate reasoning, where benefits and costs are weighed against each other and individuals choose the course of action that brings the most favorable consequences overall [5,6]. This would suggest that deviations from utilitarian moral should be more pronounced when making intuitive judgments compared to more reflective judgments. For example, actions that involve harming a single individual in order to help the many should be less likely to be deemed as morally acceptable when making intuitive judgments. An alternative theory is that intuitive and reflective moral judgments are in essence the same and that reasoning typically is used to post-hoc rationalize moral intuitions. When it comes to prosocial behavior where the decision to help others involves a cost to the self, some researchers argue that intuition leads to more altruistic behavior, while others argue that it leads to more selfish behavior.

Previous empirical studies on the role of intuition versus reflection in moral decision-making have mostly relied on relatively small sample sizes or have pooled many small-sample studies together. Moreover, a variety of experimental manipulations and outcome measures have been used. The most commonly used method to induce intuitive thinking is cognitive load. Substantially fewer studies have used time pressure.

The main objective of this study is to explore if intuitive thinking—induced by time pressure and cognitive load—influences moral judgment and altruistic behavior. More specifically we set out to explore (1) the effect of time pressure and cognitive load on judgments in trolley type moral dilemmas and (2) the effect of time pressure and cognitive load on altruistic behavior in dictator games where subjects can either keep the money for themselves or give it to charity. We conduct two experiments in three different countries (Sweden, Austria, and the United States) with in total 1,412 subjects. To the best of our knowledge this is the first large-scale study that explores the effect of time pressure in both moral judgment and altruistic behavior in an experimentally coherent way across samples. In addition, we also explore if our findings are robust when using cognitive load as an alternative way of inducing more intuitive decision making. A secondary objective of the study is to bring further knowledge regarding the existence of gender differences in moral decision-making.

### The Role of Intuition and Reflection in Moral Decision-Making

#### Moral judgment

Trolley dilemmas are a classical vehicle to explore moral judgments and the conflict between utilitarian and deontological moral foundations [7,8]. In the commonly used switch dilemma a runaway trolley is headed for five people who will be killed if it proceeds on its present course. The only way to save them is to hit a switch that will turn the trolley onto an alternate set of tracks where it will kill one person instead of five. Pulling the switch, thereby killing the single person while saving the others, is the utilitarian alternative, which implies striving towards maximizing the overall good. The non-utilitarian, deontological alternative would be not to pull the switch, since actively causing harm to another person could be considered morally unacceptable regardless of the overall consequences. When confronted with dilemmas involving conflicting moral values, is our automatic response to try to help as many as possible even if it means harming the one? Or is it to refrain from doing harm to the single individual even if it does not maximize overall benefits?

Several theories about the role of intuitive and reflective processes on moral decision-making have been proposed. A general distinction can be made between those who propose a single-process theory of moral judgment and those who advocate a dual process. Those who advocate a single-process theory can further be subdivided into those who postulate that reasoning dominates moral judgments and those who postulate that intuition dominates. Traditionally in psychology, moral judgment has been thought of as a deliberate process [9,10]. More recently, however, a stronger emphasis has been put on the dominant role of emotions in moral judgments. According to Haidt’s [11] Social Intuitionist Model, moral judgments are predominantly driven by automatic emotional responses, while moral reasoning typically is the post-hoc rationalization of intuitions. In line with the Social Intuitionist Model, individuals are often “dumbfounded” when asked to articulate underlying reasons for their moral judgments [11,12]. The general hypothesis following from a single-process approach to moral judgments would be that we should detect no difference when comparing intuitive and reflective moral decision-making. That is, moral decisions made under time pressure and cognitive load should be neither more nor less utilitarian than moral decisions made under time delay and no cognitive load, respectively.

The perhaps most influential theory for understanding how people make moral judgments has been proposed by Greene [13,14] who advocates a sequential dual-process theory of moral judgment, according to which characteristically deontological judgments are driven by automatic emotional responses, while characteristically utilitarian judgments are driven by controlled cognitive processes. Thus, cognitive processing can overturn individuals’ intuitive reactions and lead to more utilitarian judgments, see e.g., [15–17]. The general hypothesis following from this line of thought is that subjects will be less utilitarian when making moral judgments under time pressure/cognitive load. An important aspect of Greene’s theory is also that the effect of intuition should be stronger for dilemmas that involve a strong affective and personal component, such as the footbridge or lifeboat dilemmas where individuals actively have to push a person in order to save five other people.

A different type of dual process model of morality is proposed by Gürçay and Baron [18] who hold that both deontological and utilitarian responses are intuitively available but in conflict with each other. The moral judgment is a combined product of the type of person and the type of dilemma. Some dilemmas, such as the personal ones, give more strength to the deontological side while other dilemmas may give more strength to the utilitarian side. Similarly some individuals are more inclined to make deontological judgments and others are more inclined to make utilitarian judgments. Most importantly this model differs from Greene’s theory in that it does not assume any sequential effects involving suppressing an early deontological response by a later utilitarian one.

#### Altruistic behavior

A similar question regarding the role of intuition arises in situations of prosocial behavior. Is our fast intuitive response to act selfishly? Or are we predisposed towards altruistic behavior, such that reflection leads to more selfish behavior? Rand et al. [19] have proposed what they call a Social Heuristics Hypothesis (SHH), according to which prosocial behavior is typically favored through intuition, while analytical thinking adjusts behavior towards the payoff maximum in a given situation. The rationale is that prosocial behavior is typically advantageous in everyday life, leading to the formation of generalized prosocial intuitions. Thus, in one-shot anonymous interactions where selfishness maximizes individual payoff, intuitive responses should be more altruistic than deliberative responses. According to Rand et al. [20] the SHH also predicts that gender moderates the effect of intuition on altruistic behavior, so that intuition increases altruistic behavior only for women. The rationale given for this is that women are disproportionally expected to be altruistic in everyday life. Thus in a long run perspective altruistic behavior is more likely to be a payoff maximizing strategy for women when taking reputational effects into account.

In contrast to the Social Heuristics Hypothesis, Moore and Loewenstein [21] argue that self-interest is an intuitive and fast response, while understanding one’s ethical obligations to others is a more reflective process. Thus, when deciding to give money to the counterpart in a dictator game, individuals who reflect prior to the decision should give more generous offers than individuals who do not reflect prior to the decision. The general hypothesis following from this line of thought is that subjects will be less altruistic when making allocation decisions under time pressure or cognitive load.

### Previous Experiments on Intuition and Moral Decision-Making

#### Moral judgment

Most previous experiments on intuition and moral decision making have used cognitive load manipulations as a way to identify the underlying process. Although there is not a complete agreement, a majority of the existing studies on the influence of intuition indicate that intuition leads to less utilitarian moral judgment in trolley type dilemmas. Greene et al. [22] used a simultaneous digit-search task to put subjects under cognitive load, but found no effect of cognitive load on moral judgment (n = 82). Thus, cognitive load did not decrease utilitarian judgments as predicted by their dual-process model. However, they did find that, on average, cognitive load increased response time for utilitarian judgments by 0.75 seconds while no effect of cognitive load on response time was observed for deontological judgments. Greene et al. [22] argue that these results provide evidence in support of their hypothesized asymmetry between utilitarian and deontological moral judgments, with the former driven by relatively more analytical thinking and the latter by relatively more intuitive thinking. Later studies [23–25] have reported more direct evidence in support of a sequential dual process model, showing that putting subjects under cognitive load decreases utilitarian moral judgments. Moreover, Paxton et al. [17] showed that subjects became more utilitarian when primed into reflective thinking by administering the Cognitive Reflection Task [26] prior to making the moral judgments. Looking at individual traits related to intuitive and reflective thinking, Moore et al. [27] tested if individuals (n = 113) who scored high on a working-memory-capacity task were more likely to make utilitarian moral judgments. Such a finding would also be in line with the sequential dual-process model of moral judgment proposed by Greene [22]. However, their results showed no such general effect of working memory capacity on moral judgment.

Compared to cognitive load less research has explored the effect of time pressure on moral judgments in trolley type dilemmas. Suter and Hertwig [28] conducted two small-scale experiments. In the first experiment (n = 67) time-pressure was used as manipulation to invoke intuition. In the second experiment (n = 80) intuition was invoked by instructing subjects to answer questions as fast and swiftly as possible, whereas reflection was invoked by instructing them to carefully deliberate before giving an answer. Consistent with Greene et al.’s sequential dual process theory, Suter and Hertwig found that participants who were manipulated to think intuitively were more likely to give deontological responses in personal high-conflict moral dilemmas. However no effect of intuition was found for impersonal low-conflict moral dilemmas. Cummins and Cummins [29] conducted a series of experiments (total n = 508) and also found that restricting the decision time reduced the proportion of utilitarian judgments. Similar results were also found by Youssef et al. [30] who used the Trier Social Stress Test (n = 65) to explore how stress levels affect responses to personal and impersonal moral dilemmas. They found that participants who experienced the stress manipulation were less likely to make utilitarian judgments when facing personal moral dilemmas, whereas no effect of stress was found for impersonal moral dilemmas.

Correlational data related to response time may also give some insight into whether intuition leads to more deontological moral judgments (however also see Krajbich et al [31] for why it is problematic to infer intuition based on response time). Baron et al. [32] found that although response times are typically faster for deontological judgments than utilitarian judgments in personal high-conflict dilemmas such as the footbridge dilemma, no difference in response times between utilitarian and deontological judgments exists when dilemmas are difficult to resolve, that is, when subjects on average are equally likely to give a utilitarian judgment as a deontological judgment. Thus the finding that utilitarian judgments in personal high-conflict dilemmas are slower than deontological judgments can be explained in terms of decision conflict, i.e. rare responses take longer. According to a sequential dual process theory of moral judgment deontological responses should still be faster in dilemmas where utilitarian and deontological judgments are equally probable. In a meta-analysis of 26 conducted experiments where response time had been recorded Baron and Gürçay [33] provide further evidence that response time is not a reliable predictor of moral judgment—utilitarian or deontological.

#### Altruistic behavior

Turning to altruistic behavior in dictator games previous experimental research has mainly used cognitive load manipulations and response-time correlations. The effects of cognitive load manipulations on altruistic behavior in previous experiments are however ambiguous. Some studies indicate that inducing intuition through cognitive load leads to more prosocial behavior in dictator games. For instance, Schulz et al. [34] found that when playing binary dictator games (n = 136), where subjects could choose between an equal and an unequal split of money, subjects in the cognitive load condition (n-back task) more often chose the equal split. Hauge et al. [35], however, found no effect of cognitive load (memorizing a random seven digit-number) on dictator game giving when conducting a series of experiments (n = 348). Similarly Cornelissen et al. [36] also found no main effect of cognitive load on dictator game giving (n = 430), but found a treatment difference for a subset of individuals; those who were classified as prosocial in an unrelated task gave a higher amount in the cognitive load condition than in the control condition. Kessler and Meier [37] also found mixed results of the effect of cognitive load on charitable giving (n = 405). More specifically they found that when their task involving charitable giving was placed early in an experimental session cognitive load reduced charitable giving, while the opposite effect was found when the same task was placed later in the session.

When looking at response-time correlations and altruistic behavior, Cappelen et al. [38] (n = 1508) found an association between short response time and increased dictator game giving (but see also Myrseth and Wollbrant [39] for a critical note on the interpretation of these results). Piovesan and Wengström [40], on the contrary, found an association in the oppostite direction (n = 74), i.e that shorter decision time correlated with selfishness in distributive situations. It should be noted, however, that recent work by Krajbich et al [31] has showed that response-time often is driven by decision conflict/strength of preference rather than intuition versus deliberation. Thus response-time correlations may give little insight into how intuition affects moral decision making.

Rand et al [20] conducted a meta-analysis (n = 4,366) including a number of studies exploring the effects of experimentally manipulating the use of intuition versus deliberation on dictator game giving. The meta-analysis included a wide array of experimental manipulations, such as conceptual priming, cognitive load and time constrain manipulations. Most of the studies (13 out of 22) included in the analysis were previously unpublished studies from the authors’ own lab including”failed pilots, experiments with problematic design features, etc.” (p. 290). Although the authors reported no general results about intuition and altruistic behavior, they found a significant interaction between gender and cognitive processing mode. Intuitive processing made women more altruistic whereas men became more selfish, although the effect for men was significant only at the 10% level.

To sum up, the behavioral literature related to intuition and moral decision-making gives a far from coherent picture, with many results going in opposite directions. Moreover most studies have been based on small samples or pooled many small-sample studies that have used different manipulations and outcome measures.

### Gender Differences in Moral Decision-Making

The existence of gender differences in moral decision-making has been an issue of much controversy and debate. Thus an additional objective of this study is to explore gender differences in moral decision making. This additional, objective stems from the theoretical assertion that men and women use different styles of moral judgment. Based on qualitative interviews Holstein [41] has argued that women’s decisions are more frequently influenced by system 1 related processes such as empathy and emotion, while men tend to be less emotional and empathic but more impartial and detached. Similarly Gilligan [42,43] argued that men and women “speak in different moral voices”, where men predominantly are motivated by objective logic and reason, and women are guided by social emotions such as empathy and altruism. Thus, men base moral decisions on abstract principles of justice that can be universalized to every person and every situation, whereas women have more care-based orientation that emphasizes the maintenance of interpersonal relationships. However, despite numerous behavioral studies comparing moral decision-making in men and women, little evidence has been found to support Gilligan's arguments [44]. Moreover previous empirical studies on moral judgments have left gender-related differences and their relation to intuition unexplored. For dictator game giving it has been shown that women typically are more generous than men [45,46]. However, Espinosa and Kovářík [47] argue that prosocial behavior of both women and men is malleable. More specifically and highly relevant for the purpose of this study, they argue that emotional aspects and social framing tend to reinforce prosocial behavior in women but not men, whereas encouraging reflection makes men less prosocial but leaves women unaffected.

## Experimental Designs

Two experiments employing different experimental manipulations to invoke relatively more intuitive moral decision-making were conducted–*time pressure* and *cognitive load*. In both experiments subjects were randomly allocated to different experimental treatments. The experiments were run in three different countries as a between-subjects design. The total number of subjects was 1,413 (Sweden n = 510, Austria n = 320 and USA n = 583). Instructions were translated to each country’s native language, i.e. Swedish, German and English. The age range of participants was 18 to 78 years.

In both experiments subjects responded to the same four moral dilemmas presented in random order: the switch dilemma, the footbridge dilemma, the lifeboat dilemma and the fumes dilemma. These dilemmas have been commonly applied in the literature on moral psychology [14,27].

**The switch dilemma**: You are at the wheel of a runaway trolley quickly approaching a fork in the tracks. On the tracks extending to the left is a group of five railway workmen. On the tracks extending to the right is a single railway workman. If you do nothing the trolley will proceed to the left, causing the deaths of the five workmen. The only way to avoid the deaths of these workmen is to hit a switch on your dashboard that will cause the trolley to proceed to the right, causing the death of the single workman. Is it morally right to hit the switch in order to avoid the deaths of the five workmen?

**The footbridge dilemma**: A runaway trolley is heading down the tracks toward five workmen who will be killed if the trolley proceeds on its present course. You are on a footbridge over the tracks, in between the approaching trolley and the five workmen. Next to you on this footbridge is a stranger who happens to be very large. The only way to save the lives of the five workmen is to push this stranger off the bridge and onto the tracks below where his large body will stop the trolley. The stranger will die if you do this, but the five workmen will be saved. Is it morally right to push the stranger on to the tracks in order to save the five workmen?

**The fumes dilemma**: You are the late-night watchman in a hospital. Due to an accident in the building next door, there are deadly fumes rising up through the hospital’s ventilation system. In a certain room of the hospital are three patients. In another room there is a single patient. If you do nothing the fumes will rise up into the room containing the three patients and cause their deaths. The only way to avoid the deaths of these patients is to hit a certain switch, which will cause the fumes to bypass the room containing the three patients. As a result of doing this the fumes will enter the room containing the single patient, causing his death. Is it morally right to hit the switch in order to avoid the deaths of the three patients?

**The lifeboat dilemma**: You are on a cruise ship when there is a fire on board, and the ship has to be abandoned. The lifeboats are carrying many more people than they were designed to carry. The lifeboat you’re in is sitting dangerously low in the water, a few inches lower and it will sink. The seas start to get rough, and the boat begins to fill with water. If nothing is done it will sink before the rescue boats arrive and everyone on board will die. However, there is an injured person who will not survive in any case. If you throw that person overboard the boat will stay afloat and the remaining passengers will be saved. Is it morally right to throw this person overboard in order to save the lives of the remaining passengers?

Each dilemma was presented on a separate screen in written language. All moral dilemmas involved the assessment of a harmful action that maximizes good consequences and are extensively used in studies on moral judgment. The dilemmas were chosen to mirror each other with regards to the personal/impersonal distinction. Personal dilemmas involve direct bodily contact when causing harm, such as pushing a person. Impersonal dilemmas do not involve bodily contact when causing harm. Thus the footbridge and lifeboat dilemmas are personal dilemmas, while the switch and fumes dilemmas can be categorized as impersonal. Previous research has found that people are more inclined to experience emotions for personal dilemmas than for impersonal ones (see e.g. [14,16]). The footbridge and lifeboat dilemmas can also, according to the classification scheme used by Suter and Hertwig [28], be classified as “high-conflict personal” since they describe actions in which the harm involves personal force that is intentional rather than just a side effect.

**Fig 1:**
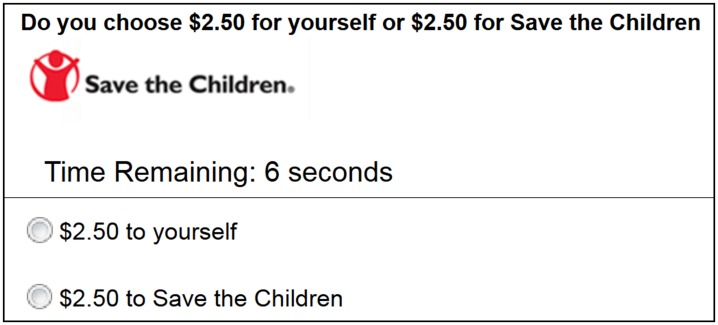
Screenshot of the binary dictator game with charitable giving.

Subjects also played four dictator games, illustrated in Fig 1, where they chose between keeping a sum of money (USA: $ 2.50, Sweden: 50 SEK (approx. $ 6.33), Austria: € 5) for themselves or giving it to a well-known charity organization (UNICEF, WWF, Save the Children, and Doctors without Borders). The dictator game is a workhorse for studying altruistic behavior in experiments since it involves no strategic concerns related to behavior. Before the session started, subjects were informed that one of the questions involving monetary payoffs would be randomly chosen for real payment after the experiment. The two experiments are described in further detail below. The complete instructions for all experiments can be found in S1 File.

### Experiment Time Pressure

In total 1,102 subjects participated in the experiment at three locations (Sweden n = 199, Austria n = 320, USA n = 583). Given the sample size we were able to detect differences (p<0.05) between experimental groups, corresponding to an effect size of Cohen’s d = 0.17 with power of 80%. In Sweden and Austria data were collected in a lab setting with student samples, recruited through email advertisement. Subjects completed the survey in a computer lab, with no interaction allowed between individuals. Data collection in USA was conducted as a web survey in collaboration with Decision Research in Eugene, Oregon. Subjects were drawn from a sample of the adult US population included in the subject pool of Decision Research.

The experiment was part of a bigger data collection investigating the effect of time pressure on economic decision-making. The complete survey was divided into five blocks of four questions each: risk taking in the gain domain, risk taking in the loss domain, a public goods game, a dictator game with charitable giving, and moral dilemmas. In addition to potential earnings from the experiment subjects also received a show-up fee of 100 SEK (€ 10 in Austria, $ 5 in the USA). Subjects were randomly assigned to one of two treatments, the time-pressure or the time-delay treatment. Treatments were identical in all aspects, except that subjects in the time-pressure treatment had to respond within 35 seconds (7 seconds in the dictator game). A timer on the screen indicated how much time they had left to respond. Subjects in the time-delay treatment had unlimited time to respond, but were required to wait 35 seconds (7 seconds in the dictator game) before any answer could be entered. All subjects responded anonymously and were informed that everyone would answer the same questions.

To test if the time-pressure manipulation was successful in inducing a difference in the degree of intuitive decision making the Jellybean task [48,49] was conducted in a separate experimental session. This task involves a hypothetical decision between two bowls containing 100 and 10 jellybeans respectively. Subjects are asked to imagine that they can draw one jellybean from one of the bowls, hidden behind a screen. If they draw a colored jellybean they win 5 Euros. The two bowls are depicted graphically with a label below the large bowl saying “9% colored jellybeans” and below the small bowl saying “10% colored jellybeans”. The more deliberative choice is to choose the small bowl because this maximizes the chances of drawing a colored jellybean, as the small bowl contains more colored jellybeans in percentage terms. However, the intuitive choice is to choose the large bowl as it contains a higher number of colored jellybeans in absolute terms. Subjects in the time pressure treatment had to answer within 7 seconds and subjects in the time delay treatment had to wait 20 seconds before responding.

### Experiment Cognitive Load

In total 311 subjects participated in the experiment at Linköping University in Sweden. Given the sample size we were able to detect differences (p<0.05) between experimental groups, corresponding to an effect size of Cohen’s d = 0.32 with power of 80% for the pooled sample. The experiment was conducted as a lab experiment with a student sample and included two separate data collections. On both occasions subjects were randomly assigned to either the cognitive load treatment or the control treatment.

In the first data collection, a common attention control manipulation was used to mentally deplete subjects in the cognitive load treatment. The paradigm involves a 7 minutes long attention control video clip (without sound) of a woman who is being interviewed by an off-camera anchor. While the woman is being interviewed, monosyllabic English words in black on a white background appear for ten seconds each in the lower quarter of the screen. Both groups saw the exact same video at the beginning of the experiment. Subjects in the control treatment received the following instructions: “The first part of this experiment involves watching a 7 minutes long video clip without sound where a person is being interviewed. You will after the movie get to answer a number of questions about the person's movements and behavior”. Subjects in the cognitive load treatment received some additional instructions: “It is of the utmost importance that you do NOT look at the words that may occur during the movie. In the event that you lose concentration from the person, return immediately to follow the person's expression". The attention control video has been used in many previous studies exploring the effect of ego-depletion on behavior [50–52]. It has also been shown to be an effective method to achieve a subjective perception of ego depletion [53] (although see [54–56]). To check if the manipulation had the predicted ego-depletion effect, subjects were asked to respond to the statement “watching the video took a lot of my energy” on a 5-point Likert scale (where 1 = *completely disagree* and 5 = *completely agree*).

The second data collection (with different subjects) was similar to the first, but in order to increase the depletion effect and avoid that the effect wears off during the experiment, an additional task was added to increase working memory load throughout the experiment. Both treatment groups were given two sequences of digits to remember while answering the dilemmas [57,58]. In the cognitive load treatment complex sequences of seven digits were shown (e.g. 7584930), while in the control treatment the sequences were simple and easy to remember (e.g., 112). Subjects who remembered the sequence correctly received 25 SEK (approximately $3.25) for each answer, i.e. participants could at most earn 50 SEK in addition to other earnings. To check if the manipulation had a depleting effect, subjects were asked to respond to the statement “remembering the digits took a lot of my energy” on a 6-point Likert scale (where 1 = *completely disagree* and 6 = *completely agree*). In addition, the formulation of the lifeboat dilemma was changed slightly compared to the first data collection, so that it no longer involved inevitable death of the person that was pushed off the lifeboat regardless of action. The lifeboat dilemma was the only dilemma that had this feature. Thus, we wanted to control for this unforeseen difference between the lifeboat dilemma and the other dilemmas.

### Ethics

We consulted the ethical review board for East Sweden to determine whether a formal approval of the committee was required. It was concluded that a formal assessment by the Ethics Committee was not necessary because the participants were given full-disclosure of the procedure (i.e., there was no deceit), participants received a payment proportionate to the task, the experimental procedure was noninvasive and the results were analyzed anonymously. Furthermore, the participants in all experiments were recruited online through our subject pools and voluntarily signed up for participation in the described experiments. They were informed participation was voluntary and anonymous. They were also informed that they could withdraw from the experiment at any time.

## Results

### Manipulation Check

Responses to the jellybean task, which was conducted in a separate experiment [59], indicate that our time-pressure manipulation was successful in inducing intuitive decisions. With time pressure 33.8% of subjects chose the larger bowl (i.e. the more intuitive choice), while only 18.3% chose the larger bowl in the time delay treatment. This effect is highly significant, chi^2^ = 10.75, *p* = 0.001, and consistent with time pressure inducing more intuitive decision-making than time delay. To test if our cognitive load manipulation was successful in inducing a difference in the degree of intuitive decision making subjects rated if the cognitive load manipulation took a lot of their energy. The average answer in the first data collection, which included only the attention control video, was 3.18 for subjects in the cognitive load treatment and 2.81 in the control treatment, *t*(113) = 1.82, *p* = 0.056. In data collection 2, where a simultaneous cognitive load task was added (7-digit task), the average answer was 3.61 in the cognitive load treatment and 1.60 in the control treatment, *t*(192) = 13.21, *p*<0.001. All subjects in the control treatment remembered both numbers correctly, while only 76% of the subjects got both numbers correct in the cognitive load treatment.

### The Effect of Time Pressure and Cognitive Load on Moral Judgment

Table 1 displays the proportion of utilitarian judgments (moral dilemmas) in each treatment. Student’s t-test was performed to test for differences between treatments for the mean rate of utilitarian judgments across all dilemmas. No significant difference between time pressure and time delay or between cognitive load and control was found. Thus, neither time pressure nor cognitive load had any general effect on moral judgment. In experiment *time pressure*, subjects in general made utilitarian judgments in 48.6% of the presented dilemmas when responding under time pressure. In the time delay treatment subjects were slightly less prone to make utilitarian judgments (mean rate 45.3%). In experiment *cognitive load*, the direction of the statistically non-significant effect of cognitive load goes in the opposite direction compared to the time pressure experiment, i.e. subjects were slightly less prone to make utilitarian judgments in the cognitive load treatment than in the control treatment.

**Table 1:** The Effect of Time Pressure and Cognitive Load on Moral Judgment.

	*Experiment Time Pressure*			*Experiment Cognitive Load*		
	Time Pressure	Time Delay	p-value	Cognitive Load	Control	p-value
n	543	559		143	168	
Female, n (%)	286 (53%)	307 (56%)	0.328	60 (42%)	70 (42%)	0.959
Age, mean	34.0	34.6	0.459	23.1	23.2	0.573
Utilitarian answers, n (%)						
Switch Dilemma	345 (66%)	327 (59%)	0.016	96 (67%)	121 (72%)	0.349
Footbridge Dilemma	132 (25%)	85 (15%)	< .001	13 (9%)	18 (11%)	0.634
Fumes Dilemma	308 (59%)	307 (55%)	0.282	98 (69%)	135 (80%)	0.017
Lifeboat1 Dilemma	240 (46%)	286 (51%)	0.060	33 (73%)	49 (70%)	0.700
Lifeboat2 Dilemma	NA	NA		23 (23%)	18 (18%)	0.380
Pooled, mean rate	0.486	0.453	0.103	0.460	0.507	0.110

For particular moral dilemmas significant effects from the time pressure manipulation were found for the switch and the footbridge dilemmas, where subjects were significantly more likely to make utilitarian judgments under time pressure than in the time delay treatment (switch dilemma: chi^2^ = 5.84, *p* = 0.016; footbridge dilemma: chi^2^ = 16.48, *p*<0.001). These findings, thus, go in the opposite direction of what would be predicted from Greene’s dual-process framework of moral judgment, i.e. that preventing controlled reasoning leads to less utilitarian judgments. However, we do not find a statistically significant difference in effect for the fumes or the lifeboat dilemmas. For experiment *cognitive load*, the experimental manipulation only had a significant effect in the fumes dilemma, where subjects were less likely to make utilitarian judgments under cognitive load than in the control treatment, chi^2^ = 5.75, *p* = 0.017.

To further explore the effect of time pressure on moral judgment, and to control for age, gender and country, logistic regression analyses were conducted with utilitarian judgments as dependent variables and treatment, age, gender, and country as predictors. An Ordinary Least Squares (OLS) regression was also conducted, in which responses from all dilemmas were pooled (using the fraction of utilitarian judgments for the four dilemmas as the dependent variable). Table 2 shows the results from these regression analyses, where the effects are presented as marginal effects (logistic regression) and betas (OLS regression). In line with the descriptive results in Table 1, we see that time pressure did not significantly predict utilitarian judgments when the moral dilemmas were pooled together, beta = .0303, *p* = .1314. Moreover, the effect of the manipulation was inconsistent across dilemmas: time pressure significantly increased the likelihood of utilitarian judgments in the switch dilemma (ME = .067, *p* = .022) and footbridge dilemma (ME = .095, *p* < .001) and decreased the likelihood of utilitarian judgments in the lifeboat dilemma (ME = -.063, *p* = .036).

**Table 2:** Logistic and OLS regressions on utilitarian judgements in moral dilemmas in the time-pressure experiment, effects shown as marginal effects (ME) and betas.

	Switch		Footbridge		Fumes		Lifeboat		Pooled	
	ME	p-value	ME	p-value	ME	p-value	ME	p-value	Beta	p-value
Treatment										
Time Pressure	0.067	0.022	0.095	< .001	0.031	0.304	-0.063	0.036	0.0303	0.131
Time Delay	REF		REF		REF		REF		REF	
Gender										
Female	-0.096	0.002	-0.103	< .001	-0.104	0.001	-0.100	0.001	-0.0992	< .001
Male	REF		REF		REF		REF		REF	
Age	0.002	0.120	0.001	0.417	0.001	0.385	-0.001	0.537	0.0009	0.388
Country		0.146		0.033		< .001		< .001		0.010
Austria	-0.015	0.421	-0.089	0.060	-0.037	< .001	-0.081	0.001	-0.0585	0.060
Sweden	-0.091	0.049	-0.065	0.524	0.179	< .001	0.131	< .001	0.0298	0.392
USA	REF		REF		REF		REF		REF	

Logistic and OLS regression analyses were also conducted for experiment *cognitive load*, with utilitarian judgments as dependent variables and treatment, gender, age, and experiment (1 or 2) as predictors. The latter was included to control for any potential effects of adding the extra cognitive load task in the second data collection and for the difference between the two versions of the lifeboat dilemma. In line with the descriptive results, Table 3 shows that cognitive load did not have a significant effect on utilitarian judgments when the moral dilemmas were pooled together, beta = -.037, *p* = .209. Moreover, cognitive load only had a significant effect on utilitarian judgments in the fumes dilemma, in which participants in the cognitive load treatment were significantly less likely to make utilitarian judgments than participants in the control treatment, ME = -.113, *p* = .023.

**Table 3:** Logistic and OLS regressions on utilitarian judgements in moral dilemmas in the cognitive load experiment, effects shown as marginal effects (ME) and betas.

	Switch		Footbridge		Fumes		Lifeboat1		Lifeboat2		Pooled	
	ME	p-value	ME	p-value	ME	p-value	ME	p-value	ME	p-value	Beta	p-value
Treatment												
Cognitive Load	-0.064	0.213	-0.019	0.583	-0.113	0.023	0.035	0.680	0.054	0.344	-0.037	0.209
Control	REF		REF		REF		REF		REF		REF	
Gender												
Female	-0.082	0.126	-0.045	0.210	0.041	0.419	-0.099	0.248	-0.123	0.050	-0.050	0.105
Male	REF		REF		REF		REF		REF		REF	
Age	0.010	0.409	0.004	0.613	-0.011	0.269	0.008	0.671	0.009	0.487	0.003	0.703
Experiment												
First	-0.166	0.003	-0.031	0.396	0.066	0.200			NA	NA	0.098	0.002
Second	REF		REF		REF		NA	NA			REF	

### The Effect of Time Pressure and Cognitive Load on Altruistic Behavior

Table 4 displays the proportion of altruistic decisions (dictator game giving) by treatment. Student’s t-test was performed to test for differences between treatments for the mean rate of altruistic decisions across all charitable causes. In line with what was found for moral judgments, there were no statistically significant effects of time pressure or cognitive load on altruistic behavior when the dictator games were pooled together. In experiment *time pressure*, 44.1% of all decisions in the time-pressure treatment were altruistic (i.e. subjects chose to give away the money instead of keeping it for themselves), compared to 46.3% in the time-delay treatment. In experiment *cognitive load*, 61.5% of all decisions made in the cognitive load treatment were altruistic, compared to 63.1% in the control treatment. Neither of these differences were statistically significant (time pressure, *t*(1100) = 0.90, *p* = 0.366; cognitive load, t(307) = 0.34, *p* = 0.734). There were no statistically significant effects of time pressure or cognitive load when choices for each charitable cause were analyzed separately.

**Table 4:** The Effect of Time Pressure and Cognitive Load on Altruistic Behavior.

	*Experiment Time Pressure*			*Experiment Cognitive Load*		
	Time Pressure	Time Delay	p-value	Cognitive Load	Control	p-value
n	543	559		143	168	
Female, n (%)	286 (53%)	307 (56%)	0.328	60 (42%)	70 (42%)	0.959
Age, mean	34.0	34.6	0.459	23.1	23.2	0.573
Altruistic answers, n (%)						
Save the Children	261 (49%)	276 (49%)	0.776	86 (61%)	103 (61%)	0.955
WWF	212 (39%)	235 (42%)	0.348	75 (53%)	95 (57%)	0.555
Doctors Without Borders	254 (47%)	269 (48%)	0.676	94 (67%)	114 (68%)	0.824
UNICEF	227 (42%)	256 (46%)	0.200	92 (65%)	112 (67%)	0.793
Pooled, mean rate	0.441	0.463	0.366	0.615	0.631	0.734

To further explore the effect of time pressure on altruistic decisions in the dictator game, and to control for age, gender, and country, logistic and OLS regressions were performed with altruistic decisions as dependent variable and treatment, gender, age, and country as predictors. Table 5 shows the results from the regression analyses. In line with the descriptive results, there was no significant effect of time pressure on altruistic decisions when all dictator games were pooled together or when decisions for each charitable cause were analyzed separately.

**Table 5:** Logistic and OLS regressions on altruistic behavior in the dictator game in the time-pressure experiment, effects shown as marginal effects (ME) and betas.

	Save the Children		WWF		Doctors Without Borders		UNICEF		Pooled	
	ME	p-value	ME	p-value	ME	p-value	ME	p-value	Beta	p-value
Treatment										
Time Pressure	-0.002	0.939	-0.020	0.481	-0.009	0.758	-0.033	0.259	-0.017	0.461
Time Delay	REF		REF		REF		REF		REF	
Gender										
Female	0.147	< .001	0.158	< .001	0.157	< .001	0.133	< .001	0.149	< .001
Male	REF		REF		REF		REF		REF	
Age	-0.001	0.497	-0.001	0.370	0.003	0.033	-0.002	0.112	-0.0002	0.883
Country		< .001		< .001		< .001		< .001		< .001
Austria	-0.348	< .001	-0.318	< .001	-0.099	< .001	-0.272	< .001	-0.256	< .001
Sweden	0.009	< .001	-0.016	< .001	0.227	< .001	0.075	< .001	0.079	0.049
USA	REF		REF		REF		REF		REF	

Regression analyses were also conducted for experiment *cognitive load*. In line with the descriptive results, Table 6 shows that cognitive load had no statistically significant effect on altruistic behavior when the dictator games were analyzed together or when they were analyzed separately.

**Table 6:** Logistic and OLS regressions on altruistic behavior in the dictator game in the cognitive load experiment, effects shown as marginal effects (ME) and betas.

	Save the Children		WWF		Doctors Without Borders		UNICEF		POOLED	
	ME	p-value	ME	p-value	ME	p-value	ME	p-value	beta	p-value
Treatment										
Cognitive Load	-0.012	0.827	-0.034	0.544	-0.015	0.779	-0.027	0.603	-0.022	0.625
Control	REF		REF		REF		REF		REF	
Gender										
Female	0.172	0.003	0.240	< .001	0.191	< .001	0.267	< .001	0.218	< .001
Male	REF		REF		REF		REF		REF	
Age	-0.004	0.740	0.023	0.065	0.010	0.403	-0.003	0.753	0.006	0.525
Experiment										
First	0.072	0.213	-0.007	0.904	-0.024	0.662	-0.111	0.046	-0.053	0.259
Second	REF		REF		REF		REF		REF	

### Gender Differences in Moral Judgments and Altruistic Behavior

**Fig 2:**
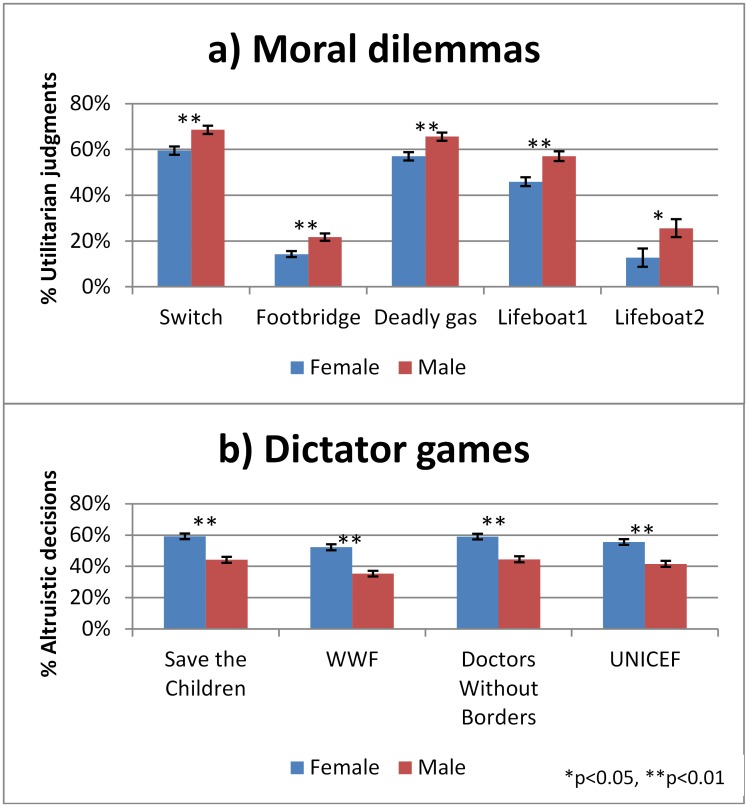
Gender differences in (A) moral judgements and (B) altruistic behavior.

Fig 2. displays utilitarian judgments and altruistic decisions for each dilemma and charitable cause, separated by gender. Males were consistently more likely to make utilitarian judgments compared to females in all dilemmas, both personal and impersonal. Across treatments and dilemmas, 53.0% of the moral judgments made by males were utilitarian, compared to 44.2% of the moral judgments made by females. A t-test shows that this difference between gender was highly significant, *t*(1401) = 5.07, *p*<0.001. In the dictator game, males were less likely to make altruistic decisions compared to females for all charitable causes. Across treatments and charitable causes, 41.3% of the decisions made by males were altruistic, compared to 56.4% of the decisions made by females. This gender difference in dictator game giving was highly significant, *t*(1400) = 6.95, *p*<0.001. However, when testing for interaction effects between gender and experimental manipulations in the pooled regressions, no significant interaction effects were detected, see S1 File Tables A-D. We furthermore tested for treatment effects separately for men and women in the pooled regressions, see S1 File Tables E-H. We found no significant treatment effects for men or women, with the exception that time pressure increased utilitarian judgments among women, see S1 File Table G. The significant effect of time pressure on utilitarian judgments among women may just be a false positive and should not carry any weight unless confirmed in other studies. The large number of tests carried out increases the risk of false positives and the result would not survive adjustments for multiple testing.

Correlational analyses were also conducted to explore the relationship between responses on the moral dilemmas and altruistic behavior. In accordance with the study by Kahane et al. [60], who used hypothetical donations, we find that greater rates of ‘utilitarian’ judgment were associated with less altruistic behavior, *r* = -0.082, *p* = .0021. This relationship held also when controlling for confounding variables (i.e. treatment, age, gender) through a partial correlation technique, *r* = -0.068, *p* = .0107.

## Conclusion and Discussion

Do individuals intuitively favor certain moral actions over others? Building on sequential dual-process theories, claims have been made that intuition should lead to more deontological moral judgments where overall consequences are disregarded. Likewise, prosocial behavior is often assumed to emerge from exerting reflective control over automatic, selfish impulses. However, recent work by for example Rand and Nowak [61] has argued that prosocial actions in the context of cooperation in the public goods game stem from intuitive processes, which was supported by the results of Rand et al [62]. However, those results failed to replicate in independent replications [63,64]. The behavioral literature related to intuition and moral judgement and altruistic behavior is also far from coherent, with effects going in both directions.

In two studies, we applied time pressure and cognitive load to investigate the effect of intuition on moral decision-making. In general, we find no effect of our manipulations on moral judgment and altruistic behavior. Thus, we find no supporting evidence for the claim that intuitive moral judgments and intuitive decisions in the dictator game differ from more reflectively taken decisions. Our results are consistent with Haidt’s [11] Social Intuitionist Model, but provide no support for Greene’s dual-process theory of morality.

A possible explanation for why we detect no difference is that intuitive processing constitutes our default mode when making moral decisions so that individuals apply automatic moral rules like “maximize life saved” and “don’t do harm” or “maximize own payoff” and “help others”. Such moral rules can be based on both consequential and non-consequential considerations and individuals thereby reconcile their intuitive and reflective thinking when forming moral rules. If so, it is also likely that moral rules are equally accessible/salient to individuals when experiencing time pressure or cognitive load. This explanation for the absence of experimental effects in our study would be in line with work by Crockett [65] and Mallon and Nichols [66,67] who have argued that dual process theories do distinguish clearly between inferred, learned and inborn responses. As Mallon and Nichols [66] state: “Dual process theory suggests that moral judgments will either be unconsciously generated intuitions or consciously available through effortful reasoning. But this neglects the possibility that there are rules that are consciously available and effortlessly applied in moral judgment” (p. 285). Following this reasoning, the formation of moral rules arguably represents moral decisions as acceptable or unacceptable in ways that are easy to employ when making decisions, thereby bypassing the finite-resources bottleneck when making moral decisions. Hence, it could be that reasoning plays a large role, but most of this important reasoning is done many years before subjects do the experiment, as part of the process of moral development.

Another possible explanation for why we detect no difference is of course that we were not successfully able to induce intuition through our experimental manipulations. We cannot know this for sure; however, time pressure and cognitive load have been standard techniques in cognitive and social psychology for experimentally manipulating the influence of intuition versus deliberation in decision-making for decades. Although ego-depletion manipulations have been called into question due to recent replication failures [56] the self-rated process measure indicates that subjects felt more energy-depleted when experiencing cognitive load. An indication that our time-pressure manipulation was successful is that we found robust behavioral effects on risk taking for losses in the same experiment [59]. Time pressure increased risk taking for losses and tended to increase risk aversion for gains compared to time delay, implying that time pressure increased the reflection effect of Prospect Theory. Moreover, the large sample size and the fact that we see the same null results using different experimental manipulations further strengthen the plausibility of our null results.

Still, the results from this study should not be taken as clear evidence against dual process theory and that moral decisions are generated either by automatic intuition or by effortful reasoning. There might still be a dual process way of thinking when making moral decisions, but the result from this study indicates that the outcomes of these processes are in essence similar. Or alternatively, that these dual processes are not sequential but parallel as it has been proposed by Gürçay and Baron [18].

One of the major factors considered in evaluating moral behavior is gender. An additional finding from this study is that we observe a significant gender gap in both moral judgment and altruistic behavior, i.e. males are more consequential in their moral judgments and less altruistic in dictator games about donations to charities. These findings are robust across all samples and all task variations and corroborate previous theory and research suggesting that men and women “speak in different moral voices” [42,43]. These findings suggest that the cognitive processes involved in moral decision making may vary between men and women, possibly reflecting differences in underlying neural mechanisms [68]. Although we see robust gender differences in moral decision making we observe no significant interactions between gender and the treatment manipulations of intuitive processing. Our experimental manipulations have no significant effects on dictator game giving for neither men nor women. This contradicts the findings from Rand et al. [20] who find that intuition favors altruism for women but not for men.

It should be noted that we in this study only explored differences between intuitive and reflective states whereas differences between intuitive and reflective personal traits were not explored. For future studies it would therefore be interesting to more thoroughly explore differences in moral decision making between individuals who may be characterized as intuitive (i.e. those who rely more heavily on intuition) and individuals who may be characterized as reflective (i.e. those who rely more heavily on reflection).

To sum up, the two experiments reported here provide converging evidence that intuitive moral decision-making does not differ from decisions made in situations where deliberation before decision is facilitated. Given the ambiguous results from the previous literature that most often has been based on small sample studies that have not been replicated and the proneness for publication bias, it is perhaps not so surprising that we find a null effect in our well-powered large sample study. The ambiguous results in previous studies may also be prone to what Gelman and Loken [69] refer to as “the garden of forking paths”, which implies lots of decisions on how to analyze the data being made after seeing the data. In line with previous studies we observe a significant gender gap in both moral judgment and altruistic behavior, i.e. males make more utilitarian moral judgments and are more selfish in the dictator game. However, there are no significant interactions between gender and the treatment manipulations of intuitive versus reflective decision-making.

## Supporting Information

S1 File Additional analyses on gender differences. Experimental instructions.
(PDF)

S2 File Data. (XLSX)
