# Supplementary material for: Intuition and Moral Decision-Making – The Effect of Time Pressure and Cognitive Load on Moral Judgment and Altruistic Behavior
Source: PLoS One. 2016 Oct 26;11(10):e0164012. doi: 10.1371/journal.pone.0164012 (PMC5082681; doi:10.1371/journal.pone.0164012)
Supplement: S1 File — Experimental instructions. (PDF) [file pone.0164012.s001.pdf]

S1 Supplementary information

1. Additional analyses on gender differences

Testing for interaction effects between gender and treatment

Table A. OLS regression, on altruistic behavior in the time pressure experiment, effects shown as betas

		Estimate	p-value
Treatment			
	Time Pressure	-0.0075	0.8263
	Time Delay	REF	
Age		-0.0002	0.8797
Country			<.0001
	AUS	-0.2561	<.0001
	SWE	0.0793	0.0495
	USA	REF	
Gender			
	Female	0.1574	<.0001
	Male	REF	
Interactions			
	Female*Time Pressure	-0.0177	0.7041

Table B. OLS regression, on utilitarian judgment in the time pressure experiment, effects shown as betas

		Estimate	p-value
Treatment			
	Time Pressure	0.0006	0.9833
	Time Delay	REF	
Age		0.0009	0.3791
Country			0.0088
	AUS	-0.0589	0.0583
	SWE	0.0301	0.3878
	USA	REF	
Gender			
	Female	-0.1265	<.0001
	Male	REF	
Interactions			
	Female*Time Pressure	0.0547	0.1749

Table C. OLS regression, on altruistic behavior in the cognitive load experiment, effects shown as betas

		Estimate	p-value
Treatment			
	Cognitive Load	-0.0167	0.7774
	Control	REF	
Age		0.0063	0.5287

Experiment			
	First	-0.0534	0.2581
	Second	REF	
Gender			
	Female	0.2239	0.0004
	Male	REF	
Interactions			
	Female*Cognitive Load	-0.0127	0.8890

Table D. OLS regression, on utilitarian judgment in the cognitive load experiment, effects shown as betas

		Estimate	p-value
Treatment			
	Cognitive Load	-0.0390	0.3135
	Control	REF	
Age		0.0025	0.7021
Experiment			
	First	0.0982	0.0017
	Second	REF	
Gender			
	Female	-0.0518	0.2087
	Male	REF	
Interactions			
	Female*Cognitive Load	0.0044	0.9412

<u>Testing for *treatment* effect for each *gender* separately</u>

Table E. OLS regression, separated by gender, on altruistic behavior in the time-pressure experiment, effects shown as betas

		Women		Men	
		Beta	p-value	Beta	p-value
Treatment					
	Time Pressure	-0.024	0.447	-0.007	0.836
	Time Delay	REF		REF	
Age		-0.0002	0.881	-0.0001	0.947
Country			<.001		<.001
	AUS	-0.290	<.001	-0.226	<.001
	SWE	0.157	0.007	0.018	0.750
	USA	REF		REF	

Table F. OLS regression, separated by gender, on altruistic behavior in the cognitive load experiment, effects shown as betas

		Women		Men	
		Beta	p-value	Beta	p-value
Treatment					
	Cognitive Load	-0.026	0.689	-0.020	0.747
	Control	REF		REF	
Age		-0.005	0.773	0.011	0.374
Experiment					

	First	-0.003	0.968	-0.093	0.160
	Second	REF		REF	

Table G. OLS regression, separated by gender, on utilitarian judgment in the time pressure experiment, effects shown as betas

		Women		Men	
		Beta	p-value	Beta	p-value
Treatment					
	Time Pressure	0.055	0.037	0.001	0.985
	Time Delay	REF		REF	
Age		0.0020	0.271	0.0001	0.968
Country			0.306		
	AUS	-0.042	0.333	-0.074	0.095
	SWE	0.021	0.672	0.034	0.494
	USA	REF		REF	

Table H. OLS regression, separated by gender, on utilitarian judgment in the cognitive load experiment, effects shown as betas

		Women		Men	
		Beta	p-value	Beta	p-value
Treatment					
	Cognitive Load	-0.034	0.444	-0.040	0.317
	Control	REF		REF	
Age		-0.003	0.817	0.0050	0.563
Experiment					
	First	0.118	0.008	0.083	0.058
	Second	REF		REF	

2. Experimental Instructions

Experiment Time pressure

Time-Pressure Treatment (changes for Time Delay treatment in brackets)

It is important that you carefully read the following instructions. The experiment consists of six parts, each part containing four to seven questions. You will in total have to answer 27 questions.

The first four parts contain questions where you will have to make decisions regarding the allocation of money. At the end of the survey, one of your answers will be drawn at random for actual payment in addition to the $5 (100 SEK) you get for participating. The last part contain questions where you will be asked to consider described situations and assess what you believe is right and wrong.

Before each new part a new set of instructions will appear on the screen. It is important that you carefully read these and that you understand the instructions before you start answering the questions.

Try to answer as quickly as possible to the questions. In the first 4 parts, you will have a maximum of 7 seconds for each question. A counter on the screen will indicate how much time you have left. If you do not respond within 7 seconds the next question will appear automatically. *(TD treatment: Think carefully through your response before proceeding. In the first 4 parts, it will take 7 seconds before you can answer each question.)*

NEW SCREEN (Dictator Game)

In this part, you have to answer four questions about how to allocate a sum of money between you and an organization.

You will choose between two distributions, one where you get all the money and one where the organization gets all the money.

NEW SCREEN

Do you choose $2.50 for yourself or $2.50 for Save the Children
Time Remaining: 7 seconds

- $2.50 (50 SEK/€5) to yourself
- $2.50 (50 SEK/€5) to save the children

(Note we used 4 different charity organizations: UNICEF, WWF, Doctors Without Borders).

NEW SCREEN (Moral dilemmas)

In the next four questions different scenarios will be described. Please answer if you think that the described action in the scenario is morally right or wrong.

You will have a maximum of 35 seconds to answer each question. *(It will take 35 seconds before you are able to provide your answer to each question)*

NEW SCREEN

You are at the wheel of a runaway trolley quickly approaching a fork in the tracks. On the tracks extending to the left is a group of five railway workmen. On the tracks extending to the right is a single railway workman.

If you do nothing the trolley will proceed to the left, causing the deaths of the five workmen. The only way to avoid the deaths of these workmen is to hit a switch on your dashboard that will cause the trolley to proceed to the right, causing the death of the single workman.

Is it morally right to hit the switch in order to avoid the deaths of the five workmen?

Time Remaining: 35 seconds

- Yes
- No

NEW SCREEN

A runaway trolley is heading down the tracks toward five workmen who will be killed if the trolley proceeds on its present course. You are on a footbridge over the tracks, in between the approaching trolley and the five workmen. Next to you on this footbridge is a stranger who happens to be very large.

The only way to save the lives of the five workmen is to push this stranger off the bridge and onto the tracks below where his large body will stop the trolley. The stranger will die if you do this, but the five workmen will be saved.

Is it morally right to push the stranger on to the tracks in order to save the five workmen?

Time Remaining: 35 seconds

- Yes
- No

NEW SCREEN

You are on a cruise ship when there is a fire on board, and the ship has to be abandoned. The lifeboats are carrying many more people than they were designed to carry. The lifeboat you're in is sitting dangerously low in the water a few inches lower and it will sink.

The seas start to get rough, and the boat begins to fill with water. If nothing is done it will sink before the rescue boats arrive and everyone on board will die. However, there is an injured person who will not survive in any case. If you throw that person overboard the boat will stay afloat and the remaining passengers will be saved.

Is it morally right to throw this person overboard in order to save the lives of the remaining passengers?

Time Remaining: 35 seconds

- Yes
- No

NEW SCREEN

You are the late-night watchman in a hospital. Due to an accident in the building next door, there are deadly fumes rising up through the hospital's ventilation system. In a certain room of the hospital are three patients. In another room there is a single patient. If you do nothing the fumes will rise up into the room containing the three patients and cause their deaths.

The only way to avoid the deaths of these patients is to hit a certain switch, which will cause the fumes to bypass the room containing the three patients. As a result of doing this the fumes will enter the room containing the single patient, causing his death.

Is it morally right to hit the switch in order to avoid the deaths of the three patients?

Time Remaining: 35 seconds

- Yes
- No

NEW SCREEN

Instructions (Jellybeans task)

On the next screen you will see two bowls. One bowl has 100 jellybeans, and the other has 10 jellybeans. You will be asked to choose one of the two bowls. Please imagine that once you have selected a bowl, it will be placed behind a screen, the experimenter will mix up the jellybeans randomly, and then you will reach around the screen (without looking at the bowl) and select a bean.

Imagine that if you selected a colored bean, you would win €5. Would you prefer to pick from bowl A or bowl B?

- [PART FOR TIME-PRESSURE]

Remember that you have a maximum of 7 seconds to answer each question. When you press 'OK' the following question will appear.

[PART FOR UNCONSTRAINED]

[empty]

- [PART FOR TIME-DELAY]

Think carefully through your response. It will take 20 seconds before you can answer the question.

Press 'OK' when you are ready to start.
NEW SCREEN

Which bowl would you prefer if a colored bean wins 5 Euros?

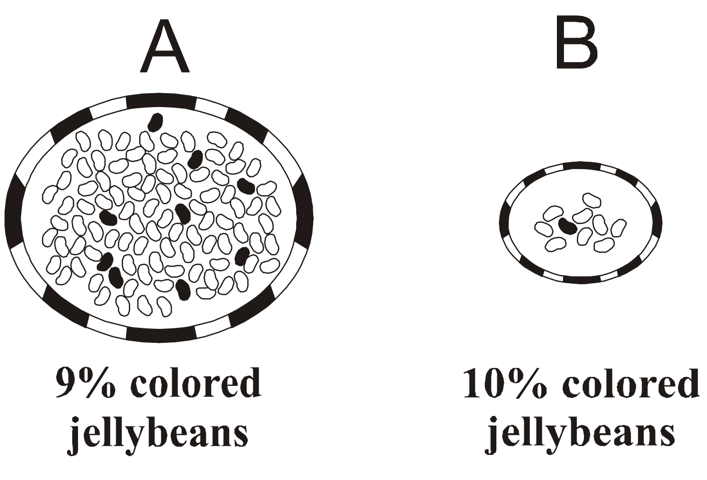

- Bowl A
- Bowl B

Experiment Cognitive load (translated from Swedish)

Cognitive Load treatment

It is important that you carefully read the following instructions. The experiment consists of three parts. You will in total have to answer 16 questions. Before each new part a new set of instructions will appear on the screen. It is important that you carefully read these and that you understand the instructions before you start answering the questions.

NEW SCREEN

The first part of this experiment involves watching a 7 minutes long video clip without sound where a person is being interviewed. You will after the movie get to answer a number of questions about the person's movements and expressions. *It is of the utmost importance that you do NOT look at the words that may occur during the movie. In the event that you lose focus from the person return immediately to follow the person's expression.* (text in italics not shown in control treatment)

Press 'next' to start the video clip.

NEW SCREEN

Please indicate if you think that the following characteristic describe the woman in the film clip

NEW SCREEN

Please indicate your level of agreement.

	Completely Agree	2	3	4	5	Completely disagree
Watching the video took a lot of my energy	❍	❍	❍	❍	❍	❍

NEW SCREEN (Only included In experiment 2)

During the next part of the experiment we want you o memorize a sequence of seven *(three)* numbers. If you report the correct sequence of numbers at the end of this part of the experiment you will earn an additional 20 SEK in payment.

The sequence of numbers that you should try to memorize is: **3856328** ***(555 in Control)***

NEW SCREEN (Dictator Game)

In this part, you have to answer four questions about how to allocate a sum of money between you and an organization.

You will choose between two distributions, one where you get all the money and one where the organization gets all the money.

NEW SCREEN

Do you choose $2.50 for yourself or $2.50 for Save the Children

- 50 SEK to yourself
- 50 SEK to Save the Children

(Note we used 4 different charity organizations: UNICEF, WWF, Doctors Without Borders)).

NEW SCREEN

Please write the sequence of numbers that you were asked to memorize

NEW SCREEN

Please indicate your level of agreement.

	Completely Agree	2	3	4	5	Completely disagree
Memorizing the sequence of numbers took a lot of my energy	❍	❍	❍	❍	❍	❍

NEW SCREEN (Only included In experiment 2)

During the next part of the experiment we want you to memorize another sequence of seven *(three)* numbers. If you report the correct sequence of numbers at the end of this part of the experiment you will earn an additional 20 SEK in payment.

The sequence of numbers that you should try to memorize is: **6807217** ***(112)***

NEW SCREEN (Moral dilemmas)

In the next four questions different scenarios will be described. Please answer if you think that the described action in the scenario is morally right or wrong.

NEW SCREEN

(All dilemmas were identical to time pressure experiment except for the lifeboat dilemma in experiment 2)

Lifeboat dilemma version 2

You are on a cruise ship when there is a fire on board, and the ship has to be abandoned. The lifeboats are carrying many more people than they were designed to carry. The lifeboat you're in is sitting dangerously low in the water a few inches lower and it will sink.

The seas start to get rough, and the boat begins to fill with water. If nothing is done it will sink before the rescue boats arrive and everyone on board will die. However If you throw one of the other persons overboard the boat will stay afloat and the remaining passengers will be saved.

Is it morally right to throw another person overboard in order to save the lives of the remaining passengers?

- Yes
- No

NEW SCREEN

Please write the sequence of numbers that you were asked to memorize
